# Lipid Melting Transitions Involve Structural Redistribution of Interfacial Water

**DOI:** 10.1021/acs.jpcb.1c06868

**Published:** 2021-11-03

**Authors:** Tereza Schönfeldová, Paulina Piller, Filip Kovacik, Georg Pabst, Halil I. Okur, Sylvie Roke

**Affiliations:** ^†^Laboratory for Fundamental BioPhotonics (LBP), ^‡^Institute of Bioengineering (IBI), ^§^Institute of Materials Science (IMX), ^∥^School of Engineering (STI), and ^⊥^Lausanne Centre for Ultrafast Science (LACUS), École Polytechnique Fédérale de Lausanne (EPFL), CH-1015 Lausanne, Switzerland; # Institute of Molecular Biosciences, Biophysics Division, University of Graz, NAWI Graz, Humboldtstrasse 50/III, Graz 8010, Austria; ¶ Department of Chemistry and National Nanotechnology Research Center (UNAM), Bilkent University, 06800 Ankara, Turkey

## Abstract

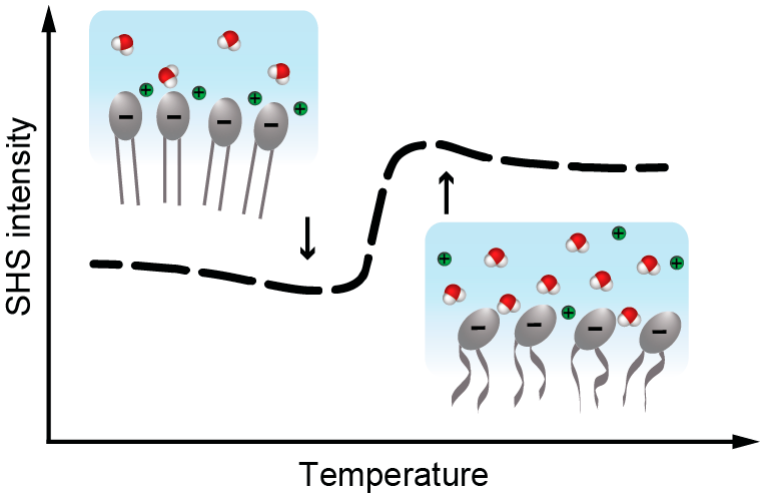

Morphological and gel-to-liquid phase transitions of lipid membranes are generally considered to primarily depend on the structural motifs in the hydrophobic core of the bilayer. Structural changes in the aqueous headgroup phase are typically not considered, primarily because they are difficult to quantify. Here, we investigate structural changes of the hydration shells around large unilamellar vesicles (LUVs) in aqueous solution, using differential scanning calorimetry (DSC), and temperature-dependent ζ-potential and high-throughput angle-resolved second harmonic scattering measurements (AR-SHS). Varying the lipid composition from 1,2-dimyristoyl-*sn*-glycero-3-phosphocholine(DMPC) to 1,2-dimyristoyl-*sn*-glycero-3-phosphate (DMPA), to 1,2-dimyristoyl-*sn*-glycero-3-phospho-l-serine (DMPS), we observe surprisingly distinct behavior for the different systems that depend on the chemical composition of the hydrated headgroups. These differences involve changes in hydration following temperature-induced counterion redistribution, or changes in hydration following headgroup reorientation and Stern layer compression.

## Introduction

Phospholipids are major building blocks of cell membranes. The diverse membranes in cells are composed of chemically diverse lipids that are present in different amounts. Membrane lipids influence the conformation and function of integral and peripheral proteins. Phospholipids are integrally involved, together with proteins and nucleic acids, in signaling cascades that control important cellular processes, including cell proliferation, apoptosis, metabolism, and migration.^1,2^ Other functions such as protein recruitment, the general permeability of the membrane to small molecules, and the mechanical properties also depend on membrane composition.^3^ The high diversity and controlled lipid composition underline the role of the biological importance of phospholipids.^4^ The structural complexity of cellular membranes is further increased by the ability of lipids to undergo phase transitions and to segregate into short- or longer-lived domains, which can be selective for either compounds or specific processes.^5,6^

Lipid phase transitions have been studied with various experimental methods including X-ray scattering,^7−10^ neutron scattering,^11−13^ nuclear magnetic resonance,^14^ electron paramagnetic resonance spectroscopy,^15−18^ fluorescence^19^ and confocal microscopies,^20,21^ FTIR measurements,^22^ and vibrational sum frequency generation spectroscopy.^23^ Theoretical studies include both coarse-grained and atomistic molecular dynamics simulations.^24−28^ Experimental and simulation studies employ various model membranes such as liposomes, supported lipid bilayers, or lipid monolayers at the air/water interface. These studies mainly report on observables that are directly related to the lipid tail properties and/or the area per individual lipid molecule. As such, the melting transition of lipids (from gel to liquid phase) has been traditionally seen as the loss of side-to-side lipid packing resulting from the increase of spacing between neighboring hydrophobic lipid tails due to intra- and intermolecular degrees of freedom.^14,22,26,29,30^ In reality, the acyl chain saturation and acyl tail length, as well as the nature of the lipid headgroups contribute significantly to the phase transition temperature.^26,29,31^ More importantly, the role of water and hydration of lipid headgroups should play an important role as well. It has been shown that exchanging H_2_O by D_2_O shifts the pretransition and main phase transition temperature.^27,32^ Although, recently, the water dynamics around lipid membranes were studied with MD simulations,^33,34^ to date the role of hydration on a phase transition remains mainly elusive. This is mostly due to the lack of sensitive experimental techniques that can probe membrane hydration in realistic freestanding bilayer systems, such as freestanding bilayer or large unilamellar vesicles (LUVs).

**Figure 1:**
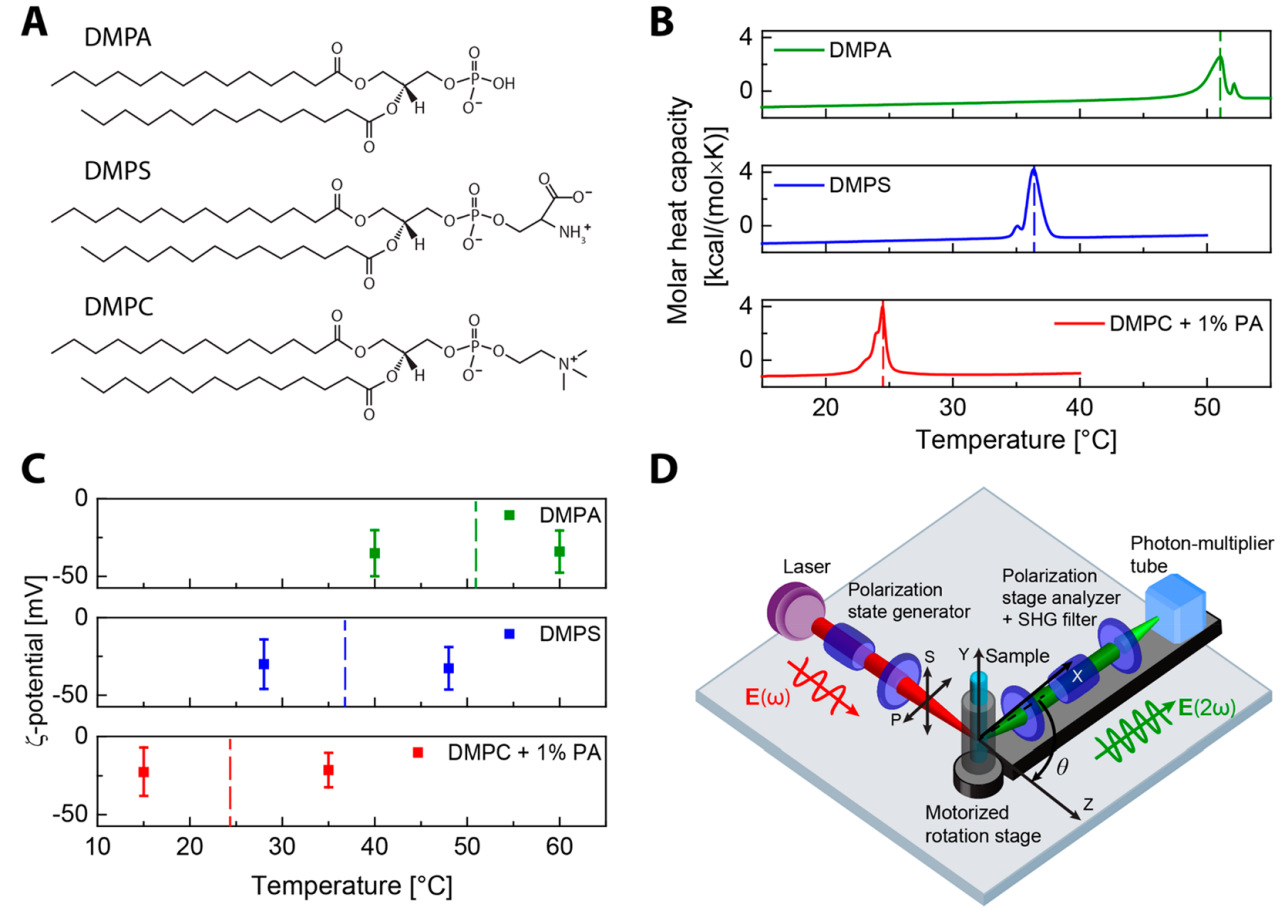
Lipid structures, DSC, and ζ-potential measurements, and the AR-SHS setup. (A) Chemical structures of lipids DMPA, DMPS, and DMPC. (B) DCS thermograms illustrating the gel-to-liquid phase transition of the single-lipid component LUVs composed of DMPA, DMPS, and DMPC with 1% DMPA. These thermograms represent the second heating scans. (C) Measured ζ-potentials of the single-lipid components LUVs composed of DMPA, DMPS, or DMPC with 1% DMPA. The dashed lines show the phase transition temperature determined by DSC measurements. (D) Illustration of the AR-SHS setup. P (S) refers to the polarization state of the beam parallel (perpendicular) to the scattering plane.

Recently, nonresonant angle-resolved second harmonic scattering (AR-SHS) was introduced, which permits the probing of the orientational order of water molecules^6,35−37^ around particle interfaces.^35,38^ In this work, we extend the study of lipid phase transitions to include structural changes in the hydration shells. We experimentally investigate the main transition of single-lipid-component LUVs made of 1,2-dimyristoyl-*sn*-glycero-3-phospho-l-serine (DMPS), and 1,2-dimyristoyl-*sn*-glycero-3-phosphate (DMPA), and 1,2-dimyristoyl-*sn*-glycero-3-phosphocholine (DMPC) with 1% DMPA (depicted in Figure 1A) in aqueous solution with differential scanning calorimetry (DSC), ζ-potential, and high-throughput AR-SHS measurements as a function of temperature. DSC measurements were performed to probe the phase transition temperatures. The interfacial water response measured by the second harmonic scattering (SHS) gave rise to a substantial increase in the orientational order of water molecules at the phase transition temperature especially for the LUVs of charged lipids (DMPS and DMPA). However, only a small increase was seen for the LUVs composed of zwitterionic DMPC lipids, although DMPC with 1% DMPA exhibits a significant second harmonic (SH) intensity increase. The underlying molecular mechanisms for the interfacial water response as captured by SHS are elucidated by theoretical modeling of the scattering patterns. By extracting the interfacial second-order susceptibility (χ_s,2_^(2)^) and surface potential (Φ_0_) of the LUVs, the interfacial structural changes were quantified. We observe that the main contribution to the SHS intensity is substantially different for different LUVs. Water response for the DMPS is influenced by both χ_s,2_^(2)^ and Φ_0_ contributions, whereas DMPA has substantial Φ_0_ contribution upon the gel-to-liquid phase transition. As such, the melting transitions influence the hydrating water molecules via different mechanisms for phospholipid LUVs with different compositions. These results demonstrate the direct link between lipid headgroup hydration, changes in surface potential, and the lipid phase transition.

## Methods

### Chemicals

Lipids 1,2-dimyristoyl-*sn*-glycero-3-phosphocholine (DMPC), 1,2-dimyristoyl-*sn*-glycero-3-phospho-l-serine (sodium salt) (DMPS), and 1,2-dimyristoyl-*sn*-glycero-3-phosphate (sodium salt; DMPA) were purchased in powder form (>99%) from Avanti Polar Lipids (Alabama, USA) and stored at −20 °C until further use. Chloroform for spectroscopy Uvasol (≥99%, Merck) and methanol (≥99.9%, Fisher Chemical) were used as received. Deconex 11 UNIVERSAL (Borer Chemie) was used as a cleaning solution. Water was purified by a Milli-Q UF-Plus instrument from Millipore, Inc., and it has an electrical resistivity of 18.2 MΩ·cm. All glassware was washed with a 5% deconex cleaning detergent solution in an ultrasonic bath for 30 min; then it was cleaned by Milli-Q ultrapure water in the sonication bath for another 20 min. After the cleaning, the glassware was rinsed with ultrapure water.

### Sample Preparation and Characterization

LUVs were prepared by the lipid film hydration method followed by extrusion. Lipid solutions were created by dissolving the 25 mg of lipid powder in chloroform in a round-bottom glass tube. To evaporate the chloroform, a gentle stream of N_2_ was directed into the rotating glass tube. The residual chloroform was dried under a room temperature vacuum for at least 3 h. The lipid film that was deposited on the glass wall was hydrated in 1 mL of ultrapure water that was heated to above the respective phase transition temperatures of the used lipids. The resulting multilamellar vesicle solutions were extruded through a 100 nm diameter polycarbonate membrane in a mini-extruder (Avanti Polar Lipids), which was preheated above the phase transition temperature of the chosen lipid. The LUVs were prepared in 150 μM Tris buffer solution at pH 7.4. LUVs were stored in closed containers for up to a week at 4 °C. The size and ζ-potential distribution of the LUVs were measured with dynamic light scattering (DLS) and electrophoretic mobility measurements (Malvern Zetasizer Nano ZS). The diameters of the LUVs for different temperatures are given in Supporting Information (SI) Table S1. The ζ-potential values are shown in Figure 1C. The concentration of the lipids in the sample was 0.5 mg of lipids/mL weight ratio for DLS, ζ-potential, and AR-SHS measurements.

### Differential Scanning Calorimetry

DSC measurements were performed using a Nano-DSC high-sensitivity differential scanning calorimeter (TA Instruments, New Castle, DE, USA). Scans of 2 mg/mL lipid concentration were recorded at a constant rate of 0.5 °C/min. Five heating/cooling cycles were conducted for each measurement. Data were analyzed using Launch NanoAnalyze (TA Instruments) including normalization for phospholipid concentration and baseline correction. The temperature at the peak maximum indicates the phase transition temperature as 51.0, 36.3, and 24.5 °C, for DMPA, DMPS, and DMPC with 1% PA, which can be compared with the literature values of 52, 35, and 24 °C for DMPA, DMPS, and DMPC.^39,40^

### Temperature-Dependent Second Harmonic Scattering

The angle-resolved second harmonic scattering setup, which enables measuring second harmonic scattering intensity at multiple angles, is depicted in Figure 1D and was previously described in ref (41). The AR-SHS measurements were performed using 190 fs laser pulses centered at 1032 nm with a 200 kHz repetition rate. The polarization state of the 1032 nm pulses was controlled by a Glan-Taylor polarizer (GT10-B, Thorlabs) in combination with a zero-order half-wave plate (WPH05M-1030). The polarized 1032 nm pulses were spectrally filtered with a long-pass filter (FEL0750, Thorlabs) and had pulse energy of 0.3 μJ, corresponding to a power of 60 mW, before the sample. They were focused into a cylindrical glass sample cell (inner diameter, 4.2 mm) down to a waist diameter of ∼55 μm and a Rayleigh length of 9.23 mm. The polarization state of the generated and scattered SH beam was analyzed (GT10-A, Thorlabs), and the spectral content was filtered with a notch filter (CT516/10bp, Chroma). The light was subsequently collimated with a plano-convex lens (*f* = 5 cm), and finally focused into a gated photomultiplier tube (PMT, H7421-40; Hamamatsu).

**Figure 2:**
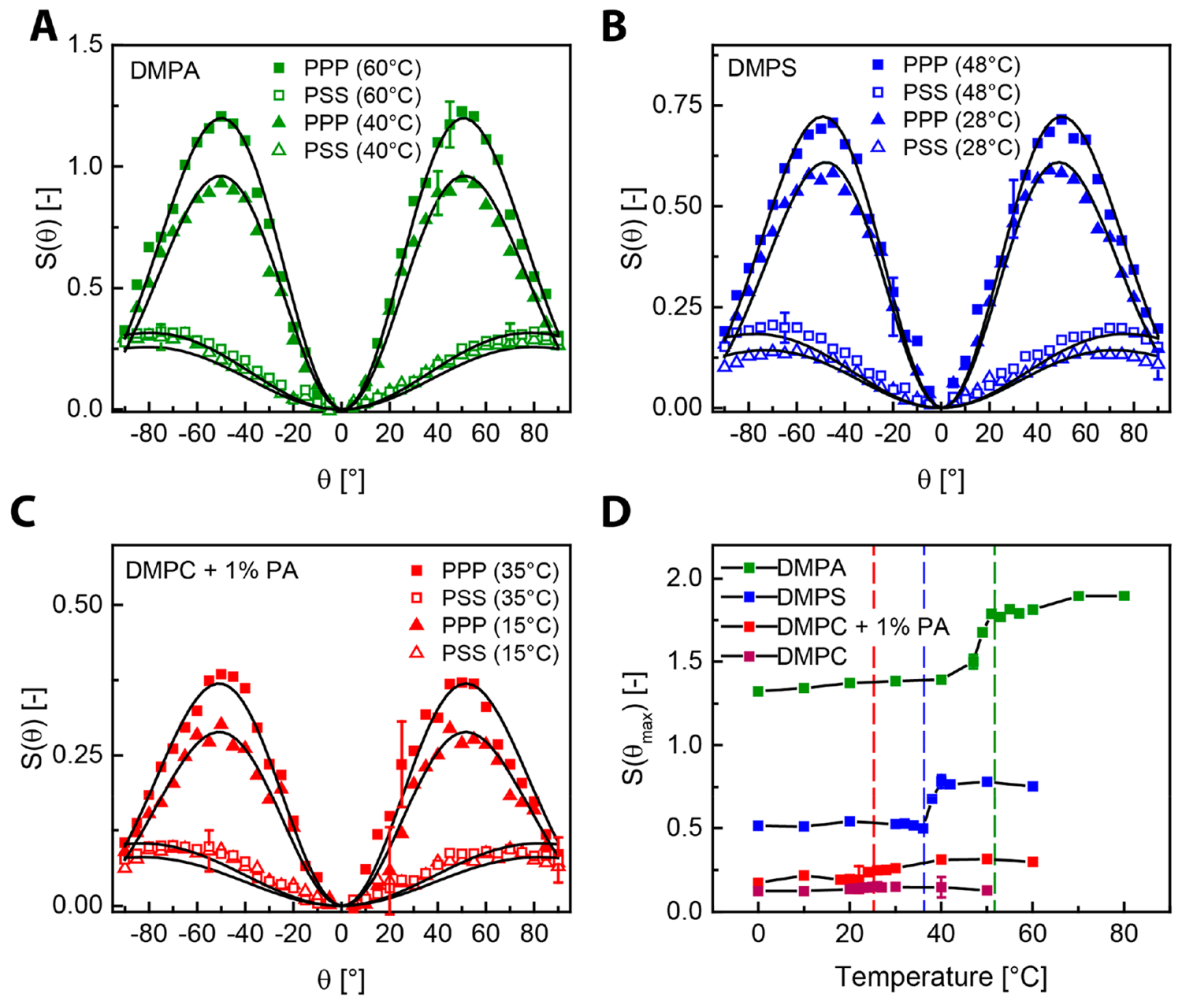
Temperature-dependent nonresonant AR-SHS measurements of LUV solutions. Nonresonant angle-resolved SHS scattering patterns for DMPA LUVs (A), DMPS LUVs (B), and DMPC + 1% DMPA LUVs (C) in aqueous solution in PPP and PSS polarization combinations. The P (S) polarization state refers to light polarized parallel (perpendicular) to the scattering angle. The PXX notation denotes the polarization of the SH beam (2ω, P), and fundamental beams (ω, S or P), respectively. Measurements were performed for a temperature 8 °C below and above the phase transition. Solid lines represent the fit to the corresponding data points using the AR-SHS model. All of the fitting parameters can be found in Table S1. (D) Single-angle (θ_max_) SHS measurements as a function of temperature from single-lipid LUVs consisting of DMPA, DMPS, DMPC, and DMPC with 1% DMPA. The dashed lines represent the phase transition temperature determined by DSC measurements. The SHS measurements were performed at the angle with the maximum SH intensity using the PPP polarization combination.

The data points for a single-angle measurement (Figure 2A) were acquired as an average of 100 measurements with a 1 s integration time and a PMT gate width of 10 ns. The detection angle, θ, which has an acceptance angle of 11.4°, was set to the angle of the maximum SH intensity (θ_max_). Scattering patterns (Figure 2B–D) were obtained by measuring SHS intensity at 5° scattering angle intervals between −90° and +90°. Each data point was acquired with an acquisition time of 20 × 1 s and a gate width of 10 ns. The angle of acceptance of the aperture before the PMT was set to 3.4°. The normalized SHS intensity at angle θ was calculated as
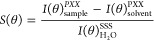
1where *I*(θ)_sample_^PXX^ and *I*(θ)_solvent_^PXX^ are the average SHS intensities of the sample and solvent at the same given temperature, respectively. *I*(θ)_H_2_O_^SSS^ is the average SHS intensity of water at room temperature. The XX stands for the polarization state of the incident beam relative to the scattering plane (P, parallel; or S, perpendicular).

To perform temperature-dependent SHS measurements, the SHS sample cell was placed in a customized temperature controller (Quantum Northwest) that provided control of the temperature of the sample: The temperature was tunable from −253.15 K (−20 °C) to 423.15 K (150 °C) with a precision of ±0.1 K. All measurements were performed in a temperature- and humidity-controlled room (*T* = 297 K; relative humidity, 26.0%).

### Fitting the AR-SHS Patterns

The normalized AR-SHS patterns in PPP and PSS polarization combinations were fitted to determine the values of the second-order susceptibility and surface potential using formalism previously described elsewhere.^42^ The following parameters were used: refractive indices of water (1.33) and LUVs (1.45), SH wavelength 516 nm, the respective temperature of the sample, the radius of the LUVs and the ionic strength (determined from conductivity measurements), and the number of particles per mL. All experimental parameters used for the fitting are summarized in Table S1.

## Results and Discussion

### Characterizing Lipid Phase Transitions in LUVs

LUVs prepared from the lipids shown in Figure 1A with diameters in the range 93–118 nm were formed by film hydration and subsequent extrusion. Details of the preparation can be found in Methods, and sample characteristics are given in Table S1. Heating differential scanning calorimetry thermograms of single-lipid LUVs of DMPA, DMPS, and DMPC with 1% of DMPA in aqueous solution are shown in Figure 1B. The peaks observed in the thermograms correspond to the lamellar gel-to-liquid phase transition which occurs at 51.0, 36.3, and 24.5 °C, for DMPA, DMPS, and DMPC with 1% PA, respectively. The differences in phase transition temperature (e.g., 26.5 °C between DMPC and DMPA) between the different DM lipids in Figure 1B demonstrate various interactions involving other parts besides the acyl chains. One important influence is the zwitterionic (PC) versus the charged nature of the lipid headgroups (PA and PS), and it is clear that the ionization state of the lipids can influence the phase transition temperature, as it influences the interactions between the headgroups. Another major component of the lipid bilayer is the hydrating water. The hydrogen bonding capacities of PA and PS headgroups are higher than that for PC. The main phase transition temperatures can also be influenced by interlipid hydrogen bonds^43^ that may form in the DMPA^44^ and DMPS^45^ bilayer, further increasing the difference from the phase transition temperature of DMPC.

The effect of charge can be investigated using electrokinetic mobility measurements that report on the mobility of LUVs in an aqueous solution. The measured mobility can be converted into a ζ-potential value, which is the converted potential at the slip plane.^46^Figure 1C shows ζ-potential values of the same LUVs solutions of each lipid at temperatures at least 8 °C above and 8 °C below the phase transition temperature. The phase transitions determined by the DSC measurements of Figure 1A are denoted by the dashed lines in the figure. The ζ-potential values of the LUVs are seen to be independent of temperature and have values of ζ = −35.1 ± 14.9 mV for DMPA, ζ = −30.0 ± 16.0 mV for DMPS, and ζ = −22.6 ± 15.4 mV for DMPC, respectively. Although the ζ-potential is a good way of obtaining an indication of the sign of the charge, it does not provide a quantitative measure of the electrostatic environment of the electric double layer of the LUV as it is an indirect measurement at an undefined location. Indeed, recent studies of the electrostatic potential and double-layer environment of LUVs,^35,38^ silica particles,^47^ and titania particles^48^ in aqueous solutions have shown that the ζ-potential is not a very accurate indicator of the surface potential. A more accurate way to determine the surface potential and interfacial water structure is to use nonresonant angle-resolved second harmonic scattering.

### LUV Hydration Quantified

In a nonresonant AR-SHS experiment a pulsed femtosecond near-infrared beam interacts with a LUV solution. The experimental setup is displayed in Figure 1D. Coherent second harmonic photons are emitted from nonisotropic molecules in the nonisotropic interfacial region of the LUVs. The nonresonantly generated SH photons originate from all dipolar molecules that are noncentrosymmetrically distributed. The emitted SH field from each dipolar molecule has the same order of magnitude.^49^ In the interfacial electric double-layer region, water outnumbers lipids in a ratio of 1:50 or more. Since the SH intensity scales quadratically with the surface density^50^ the scattered SH photons generally report on the water in the interfacial region. Therefore, the SH intensity reports on the net orientational order of interfacial water molecules along the surface normal, induced by either electrostatic or other nonelectrostatic interactions (such as hydrogen bonding and van der Waals interactions).

The SHS intensity *I*_2ω_ is expressed as the absolute square of the sum of a term that reports on electrostatic field induced interactions (Γ^(3)′^ term) and all other interactions (Γ^(2)^ term):^42,51^
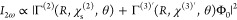
2where *R* is the LUV radius, χ_s_^(2)^ is the second-order surface susceptibility, θ is the scattering angle, χ^(3)′^ is the effective third-order surface susceptibility (composed of a number of terms^38^), Φ_0_ is the surface potential, and Γ^(2)^ and Γ^(3)′^ are second-order and third-order particle susceptibilities, respectively. As mentioned, the water molecules in the interfacial region can be oriented in two ways: By electrostatic field interactions in the case of charged surfaces, or by all other chemical interactions confined to the membrane surface. The first of the two contributions are directly related to Γ^(3)′^, which is quantified by the surface potential, and the second part of the contribution reflects on changes in Γ^(2)^ that contains the interfacial second-order susceptibility (χ_s,2_^(2)^), which reports on the average orientational distribution of water molecules in the direction of the surface normal.^38,42,52^

### Hydration Structure above and below the Phase Transition

AR-SHS scattering patterns of DMPA, DMPS, and DMPC with 1% of DMPA solutions 8 °C below and 8 °C above their corresponding phase transition temperature are shown in Figure 2A–C, respectively. Patterns were measured in PPP and PSS polarization combinations. The black lines correspond to fits made by nonlinear light scattering theory that allows extracting Φ_0_ and χ_s,2_^(2)^ of water. It can be seen that all three different LUV systems generate AR-SHS patterns with different temperature-dependent changes. This clearly shows that the hydrated headgroup region participates in the phase transition and that besides conformational changes in the acyl chains also the hydration around (mostly) the headgroups experiences significant structural changes.

To investigate these structural changes in the hydrated layer further, Figure 2D shows fixed angle SHS measurements of single-lipid LUVs solutions as a function of temperature using the PPP polarization combination. The data are collected at the maximum scattering angles (θ_max_) of Figure 2A–C, showing maximum intensities of DMPA (green), DMPS (blue), DMPC + 1% DMPA (red), and pure DMPC LUVs (black) in aqueous solution. The phase transition temperature as measured by DSC (T_DCS_) is indicated by a vertical dashed line. For DMPA LUVs, the SHS intensity increases by 31% between the temperature below (40 °C) and above (60 °C). Also, the rapid increase in the SH intensity (*T*_SHS_) initiates 4 °C below the DSC phase transition temperature. For DMPS, the change in the SH intensity from below (30 °C) to above (50 °C) the phase transition is 49%. Here, the SH intensity increment starts after the phase transition temperature, so that *T*_DCS_ < *T*_SHS_. The difference in onset temperature between DMPA and DMPS LUVs hints at different molecular mechanisms for the SHS intensity rise on phase transition. Since the SHS intensity jump for DMPA LUVs occurs below that of the main phase transition temperature, while for DMPS it occurs later, it suggests that the water reorientation initiates the phase transition in the case of DMPA and it follows the transition in the case of DMPS. It also implies that for DMPS the conformational changes of the acyl tails enable the hydration transition. For pure DMPC no temperature-dependent change is observed for the maximum SH intensity. When 1% of DMPA is introduced to DMPC, the SH intensity enhancement alters from 10% to 33%. Such significant rises of the SHS intensity around the main transition temperature for all of the studied LUVs demonstrate a clear reorientation of interfacial water molecules around the lipid headgroups.

**Figure 3:**
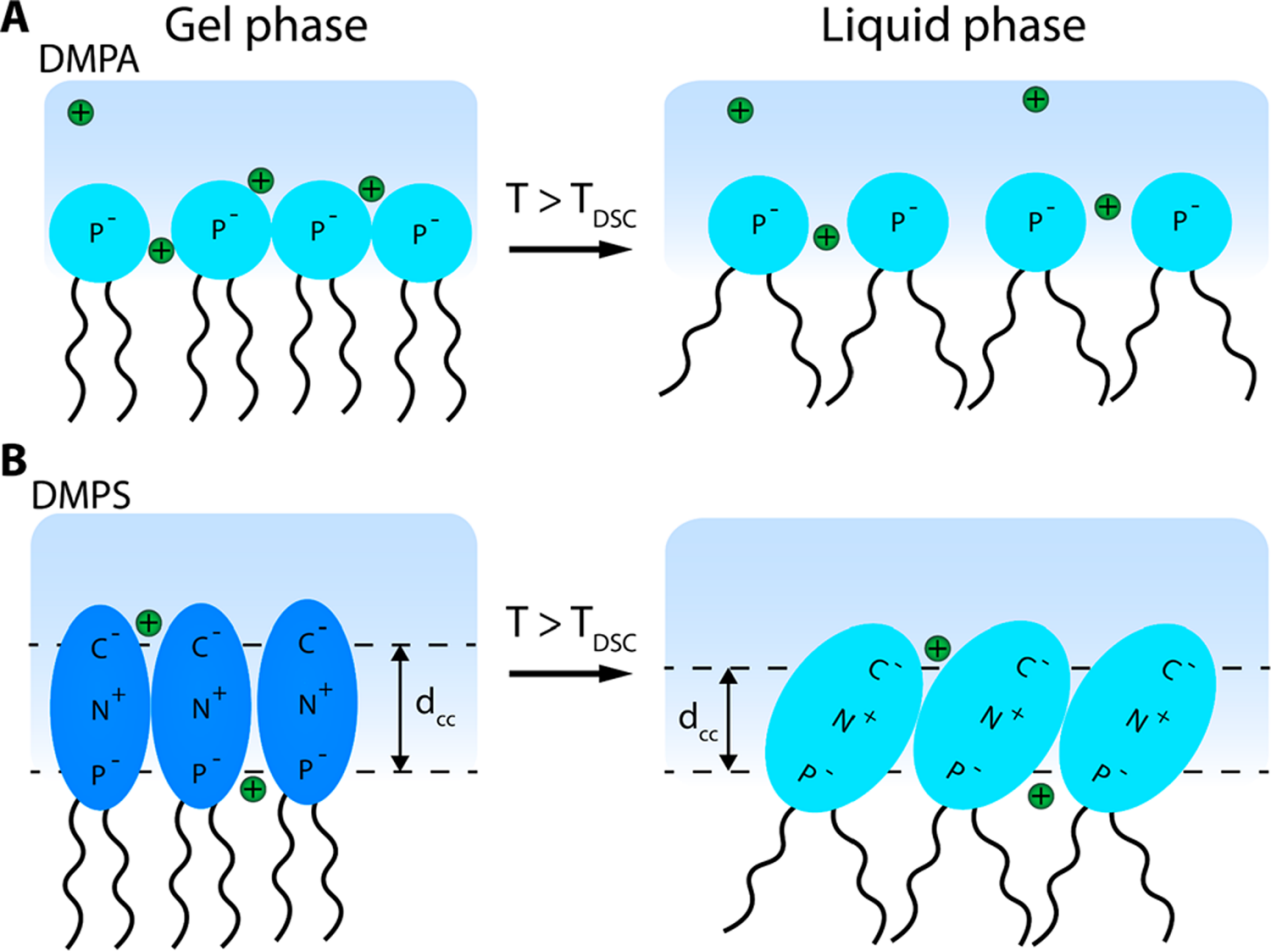
Illustration of the structural changes in hydration between the gel and liquid phases of DMPA and DMPS LUVs. (A) No DMPA headgroup reorientation observed during the phase transition. Thus, the hydration of the PA headgroup remains similar. However, the interfacial water molecules are reoriented by the increase of the surface potential due to the counter cations dissociation. (B) DMPS headgroups can reorient upon the phase transition increasing its hydration. The thickness of the charge condensation layer (*d*_cc_) is decreasing upon the phase transition. For simplicity the charged groups are shown by single-atom notation, and the explicit chemical headgroup structure can be seen in Figure 1A.

To quantify the observed changes in the SHS intensity that corresponds to the changes in the amount and orientation of the interfacial water, we modeled the AR-SHS patterns with nonlinear light scattering theory to retrieve Φ_0_ and χ_s,2_^(2)^. The procedure is discussed in the Methods and can be found in detail in ref (42). All results are summarized in Table 1, and all experimental parameters used for the fitting are tabulated in the SI. Comparing the AR-SHS patterns in PPP and PSS polarization combinations for DMPA LUVs below and above the phase transition (Figure 2A), the SHS intensity is seen to increase significantly in the PPP polarization combination with the phase transition temperature. However, there is no detectable change in the PSS polarization combination when undergoing the phase transition. The SHS intensity in the PPP polarization combination is more sensitive to changes in Φ_0_, while the PSS polarization combination is influenced more by the changes in χ_s,2_^(2)^.^38^ Furthermore, DMPA (Figure 1A) has a very small and symmetric headgroup that contains only the negatively charged phosphate group together with a Na^+^ counterion. On the basis of this structure, one can expect that the DMPA headgroup does not undergo any reorientation during the phase transition. We expect minimal interfacial water reorientation due to headgroup reorientation, and the main changes in the AR-SHS patterns are likely arising from changes in the interfacial electrostatics (i.e., counterion distribution) and not from the χ_s,2_^(2)^ contribution, consistent with the observation that the PPP intensity is temperature-dependent, while the PSS intensity is not. Therefore, a global AR-SHS fit was made to the four scattering patterns, taking the ζ-potential (−35.1 mV) as a starting point for the surface potential and allowing a 10% change in the χ_s,2_^(2)^ value. This resulted in a value of χ_s,2_^(2)^ = (2.1 ± 0.2) × 10^–22^ m^2^ V^–1^ for both temperatures, while the magnitude of the surface potential increased from −35 mV to Φ_0_ = −56 ± 9 mV. This trend is expected, since a temperature change also affects the Debye screening length (see SI), leading to an overall increase in the magnitude of the surface potential. Thus, with water reorientation due to counterion condensation, we can explain the observed changes in the nonresonant AR-SHS patterns. This structural change is illustrated in Figure 3A.

**Table 1:** Recorded Temperatures and AR-SHS Fit Parameters

	*T*_DSC_ (°C)	*T*_SHS_ (°C)	*T* (°C)	Φ_0_ (mV)	χ_s,2_^(2)^ (10^–22^ m^2^ V^–1^)^a^
DMPA	51.0	47.9	40	–35 ± 0	2.1 ± 0.2
			60	–56 ± 9	2.1 ± 0.2
DMPS	36.3	37.6	28	–90 ± 15	0.4 ± 0.1
			48	–50 ± 13	1.3 ± 0.7
DMPC + 1% DMPA	24.5	23.2	15	–23 ± 0	1.5 ± 0.4
			35	–34 ± 8	1.5 ± 0.4

a Note that the convention on the χ_s,2_^(2)^ positive sign means that the interfacial water molecules have a net orientation pointing toward the surface with their H atoms.^53^ For a negative sign the orientation is reversed.

Figure 2B shows AR-SHS patterns for an aqueous solution of DMPS LUVs recorded in the PPP and PSS polarization combinations for temperatures 8 °C above and below the phase transition. In this case, the AR-SHS patterns increase in intensity above the phase transition temperature for both polarization combinations. Therefore, on the basis of the changes in the AR-SHS patterns, changes in both Φ_0_ and χ_s,2_^(2)^ are expected. Keeping both parameters free in the global fits, we obtained as interfacial fit parameters: Φ_0_ = −90 ± 15 mV and χ_s,2_^(2)^ = (0.4 ± 0.1) × 10^–22^ m^2^·V^–1^ values, and for the temperature above the phase transition the fitting yielded values of Φ_0_ = −50 ± 13 mV and χ_s,2_^(2)^ = (1.3 ± 0.7) × 10^–22^ m^2^·V^–1^. Thus, we observe an increase in the magnitude of the χ_s,2_^(2)^ value and a decrease in the magnitude of the surface potential. Unlike DMPA, DMPS lipids have a larger and chemically more complex headgroup. However, these data can be understood if we start with the structural information that is known about PS monolayers and bilayers. DOPS LUVs were recently characterized to have a charge condensation or Stern layer, even at very low ionic strengths.^35,38^ Furthermore, condensed phase DPPS monolayers were formed on the surface of 100 nm oil droplets that were suspended in aqueous solution and characterized by vibrational sum frequency scattering and second harmonic scattering.^54^ It was found that for a condensed acyl chain structure the headgroups are oriented with the phosphate dipole oriented along the surface plane, leading to a P–N dipole in the direction of the surface normal. This is a structure that is consistent with a minimal headgroup area that befits a tight packing. We can assume that for the DMPS bilayer, below the phase transition, the headgroup will have a similar structure, and there will be a charge condensation layer as well. Note that the latter is confirmed by the difference in magnitude of the ζ-potential and the surface potential.^47^ Increasing the temperature above the phase transition temperature (*T*_DSC_), the acyl chains will occupy more space with an increasing number of chain defects. This leads to more space for the PS headgroups, suggesting that the headgroups will have more orientational freedom with bigger tilt angles away from the surface normal. Such an increase in tilt angle leads to a larger number of associated hydrating water, resulting in an increase in χ_s,2_^(2)^. Additionally, the reduction in surface potential is explained by the concomitant reduction in the charge condensation or Stern layer thickness, which leads to a smaller surface potential value. These structural transitions are illustrated in Figure 3B, where *d*_cc_ denotes the thickness of the charge condensation layer.

In the next set of experiments, LUVs containing zwitterionic lipids, DMPC, were measured. These data for pure DMPC LUVs show that the SHS intensity is changing neither in the PPP nor in the PSS polarization combination (see Figure S1). This indicates that upon phase transition χ_s,2_^(2)^ and Φ_0_ remain the same. In this case, no significant reorientation of water molecules at the interface was observed. Indeed, PC headgroups of condensed DPPC monolayers around oil droplets in water were also studied with vibrational sum frequency scattering. The PC headgroups were found to have a nearly perpendicular orientation compared to the PS headgroups, which is driven by electrostatic interactions between neighboring headgroups.^54^ At temperatures above the phase transition it is possible that the headgroups will have less overlap and therefore a little more hydration, but this hydrating water will be oriented mostly in the interfacial plane and therefore does not lead to an increase in χ_s,2_^(2)^, which only reports on water that has a (partial) dipole orientation parallel to the surface normal.

Finally, we investigated a LUV sample having 1% of DMPA in DMPC. Such vesicles were made to mimic cell membranes that include sparsely negatively charged lipids. Figure 2C shows a rise in the intensity of the AR-SHS patterns for the PPP polarization combination upon the phase transition. Yet, there is no measurable change in the PSS polarization combination. Using the same reasoning as for the DMPA LUVs, a global AR-SHS fit was made to the four scattering patterns, taking the ζ-potential (−22.6 mV) as a starting point for the surface potential and allowing a 10% change in the χ_s,2_^(2)^ value. This resulted in a value of χ_s,2_^(2)^ = (1.5 ± 0.4) × 10^–22^ m^2^·V^–1^ for both temperatures, while the magnitude of the surface potential increased from −22.6 mV to Φ_0_ = −34 ± 8 mV. Therefore, also in this case the changes in the SH intensity primarily arise from counterion motion, while the hydration shells are not changed in size.

### Membrane Hydration Comparison

Having described the temperature response of the three different LUVs systems, we note that each system behaves in a different way, caused by the plethora of interactions that are playing different roles in phospholipid–water–ion interactions. It cannot be expected that water adjacent to these different lipid membranes behaves in the same way. In contrast, the hydrophobic cores of the different lipid membranes undergo a similar melting transition.

For DMPC with in-plane-oriented headgroups very little change in the hydration is observed. Since AR-SHS is only sensitive to the molecular orientation of the water in the radial direction, we cannot conclude that there is no change in hydration as there might be changes in the surface plane that are not detected. There is, however, a clear difference between DMPC, and DMPA and DMPS LUVs. In the case of DMPA LUVs, the headgroup hydration remains unchanged owing to its small size. Here, a temperature-dependent increase in the interfacial water ordering is observed due to countercations dissociation, as illustrated in Figure 3A. DMPS on the other hand has a larger and more complex hydrated headgroup structure. During the phase transition of the hydrophobic core, the space that is available for the lipid headgroups increases. This leads to a reorientation of the hydrated headgroups, which changes the water structure. This reorientation also reduces the thickness of the charge condensation or Stern layer, leading to a reduction in surface potential. These structural changes are illustrated in Figure 3B.

Overall, our observations provide powerful experimental evidence that the main transition of lipid bilayers does not only involve melting/crystallization of the hydrophobic core but also involves the complex interactions of the hydrated headgroup region. The spatial extent of the headgroups, the counterion condensation, hydrogen bonding, and other interactions are all relevant, and these lead to different types of responses and structural rearrangements. This means that the complexity of lipids in the cell membrane might well be tuned to not only optimize the conditions inside the membrane but also to tune properties of the adjacent aqueous environment, as was recetly hypothesized.^4^ Recent measurements of the dynamic structural changes in hydrated bilayers^55,56^ support this view and demonstrate the need for further research.

## Conclusions

In summary, in this work, we probed structural changes in the hydration of single-lipid-component LUVs made of pure DMPC, and DMPA, DMPS, and a mixture of DMPC with 1% DMPA that accompanied the well-known lipid main transitions. Differential scanning calorimetry (DSC), ζ-potential, and AR-SHS measurements were performed as a function of temperature. The DSC measurements accurately determined the phase transition temperature. The ζ-potential measurements showed no apparent change of the charge at the slipping plane. The temperature-dependent SHS experiments showed substantial changes that were different for the different LUVs. Theoretical modeling of the AR-SHS provided values for the two contributors to the SHS intensity, the interfacial second-order susceptibility (χ_s,2_^(2)^) and the surface potential (Φ_0_). Surprisingly, considerably different behaviors are found for different LUVs. DMPA LUVs solely display surface potential changes that accompany the gel-to-liquid phase transition, whereas DMPS LUVs display changes in both χ_s,2_^(2)^ and Φ_0_. DMPC shows no apparent changes in either of the contributions, although DMPC with 1% DMPA exhibits an increase in Φ_0_.

Our data demonstrate the direct link between lipid headgroup hydration, changes in surface potential, and the lipid phase transition. Given that the strength of interactions in the headgroup interfacial region is generally larger than those in the hydrophobic core, we expect that these need to be incorporated when considering membrane transitions.

## Supporting Information Available

Angle-resolved SHS scattering patterns for DMPC LUVs in PPP and PSS polarization combinations below and above the melting transition; table with AR-SHS fitting parameters; equation showing the dependence of Debye length on the temperature (PDF)
