# Supplementary material for: Lipid Melting Transitions Involve Structural Redistribution of Interfacial Water
Source: J Phys Chem B. 2021 Nov 3;125(45):12457–65. doi: 10.1021/acs.jpcb.1c06868 (PMC8607985; doi:10.1021/acs.jpcb.1c06868)
Supplement: Supplementary file 1 — jp1c06868_si_001.pdf [file jp1c06868_si_001.pdf]

Supplementary information for

Lipid Melting Transitions Involve Structural Redistribution of Interfacial Water

Tereza Schönfeldová[1], Paulina Piller[2], Filip Kovacik[1], Georg Pabst[2], Halil I. Okur[1,3] and Sylvie Roke[1,*]

[1]*Laboratory for fundamental BioPhotonics (LBP), Institute of Bioengineering (IBI), and Institute of Materials Science (IMX), School of Engineering (STI), and Lausanne Centre for Ultrafast Science (LACUS), École Polytechnique Fédérale de Lausanne (EPFL), CH-1015 Lausanne, Switzerland*

[2]*Institute of Molecular Biosciences, Biophysics Division, University of Graz, NAWI Graz, Humboldtstr 50/III, Graz, 8010, Austria*

[3]*Department of Chemistry and National Nanotechnology Research Center (UNAM), Bilkent University, 06800 Ankara, Turkey*

* *sylvie.roke@epfl.ch*

S1: SHS measurements of DMPC LUVs

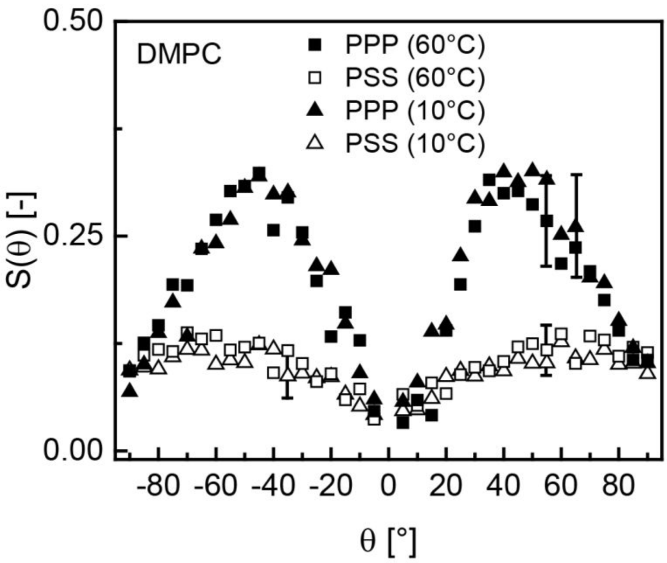

Figure S1: SHS measurements of DMPC LUVs aqueous solution above and below its gel to liquid phase transition. Angle-resolved SHS scattering patterns for DMPC LUVs in PPP and PSS polarization combinations below and above the phase transition.

S2: AR-SHS fitting parameters

Table S1: AR-SHS fitting parameters. T is a temperature, d is a z-average diameter of the LUVs obtained from DLS measurements, PDI is the polydispersity index of the LUVs obtained from the DLS measurements, c is the ionic strength of the LUVs obtained from conductivity measurements (for calculations see ref.[1,2]) and N_p is the number of LUVs per mL for 0.5 mg/mL solutions.

Lipid type	T [°C]	d [nm]	PDI [-]	c [M]	N_p [#/mL]
**DMPA**	40	107.1 ± 0.4	0.04 ± 0.01	3.71E-04	3.78E+12
**DMPA**	60	115.6 ± 0.4	0.007 ± 0.008	3.71E-04	3.78E+12
**DMPS**	28	118.4 ± 0.4	0.07 ± 0.01	3.57E-04	2.92E+12
**DMPS**	48	116 ± 2	0.062 ± 0.008	3.57E-04	2.92E+12
**DMPC + 1 % DMPA**	15	92.29 ± 0.07	0.06 ± 0.01	1.50E-04	4.35E+12
**DMPC + 1 % DMPA**	35	105.3 ± 0.5	0.04 ± 0.01	1.50E-04	4.35E+12

S3: Debye length

Debye screening length (κ^{-1}) for monovalent electrolyte is described by the following equation :

$$\kappa^{-1} = \sqrt{\frac{\varepsilon_0 \varepsilon_r k_B T}{2 N_A e^2 I}}, \qquad \text{(S1)}$$

where ε_0 is the permittivity of vacuum, ε_r is the relative permittivity, k_B is Boltzmann constant, T is the absolute temperature, N_A is the Avogadro number, e is the elementary charge and I is the ionic strength.

References

(1) Marchioro, A.; Bischoff, M.; Lütgebaucks, C.; Biriukov, D.; Předota, M.; Roke, S. Surface Characterization of Colloidal Silica Nanoparticles by Second Harmonic Scattering: Quantifying the Surface Potential and Interfacial Water Order. *The Journal of Physical Chemistry C* **2019**, *123* (33), 20393–20404. https://doi.org/10.1021/acs.jpcc.9b05482.

(2) Bischoff, M.; Biriukov, D.; Předota, M.; Roke, S.; Marchioro, A. Surface Potential and Interfacial Water Order at the Amorphous TiO_2 Nanoparticle/Aqueous Interface. *J. Phys. Chem. C* **2020**, *124* (20), 10961–10974. https://doi.org/10.1021/acs.jpcc.0c01158.
